# Kasai-like repair of recurrent pulmonary venous obstruction

**DOI:** 10.1016/j.xjtc.2024.10.025

**Published:** 2024-11-23

**Authors:** Majid Husain, Thayne Dalrymple, Megan Schultz, Ashley Prosper, Daniel Levi, Glen Van Arsdell

**Affiliations:** a Division of Pediatric Cardiology, Department of Pediatrics, David Geffen School of Medicine at UCLA, Los Angeles, Calif; b Division of Pediatric Critical Care, Department of Pediatrics, David Geffen School of Medicine at UCLA, Los Angeles, Calif; c Division of Cardiac Surgery, Department of Surgery, David Geffen School of Medicine at UCLA, Los Angeles, Calif; d Department of Radiological Sciences, David Geffen School of Medicine at UCLA, Los Angeles, Calif

**Figure:**
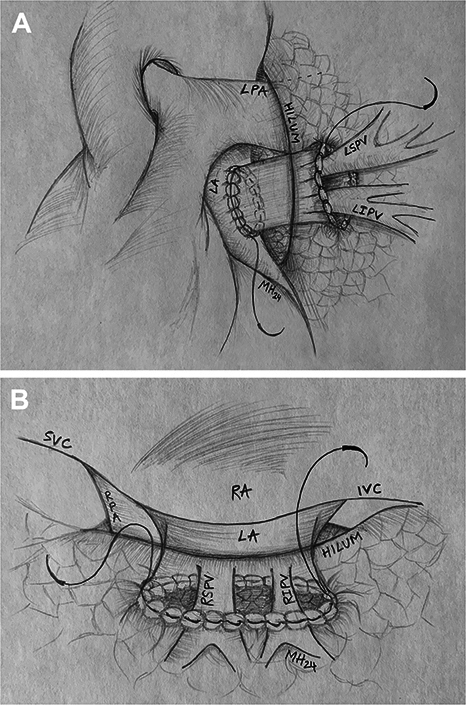
A lung hilum Kasai-like anastomosis of atrium or conduit for hilar PVO.

**Central Message**

A lung hilum Kasai-like anastomosis, of either the atrium or a conduit, is a feasible option for repair of hilar pulmonary vein obstruction in patients with recurrent and distal PVO.

Pulmonary venous obstruction (PVO) remains a major complication and the main cause of reoperation after repair of total anomalous pulmonary venous return (TAPVR).^1^^,^^2^ Although a primary sutureless repair is preferred to address this pathology,^3^ execution can be limited by distant PVO. We describe a repair for hilar pulmonary vein obstruction in 2 patients with infradiaphragmatic TAPVR. The repair is analogous to the Kasai repair for biliary atresia. The institutional review board (IRB) approved the study protocol (UCLA IRB #22-000385; approved March 18, 2022) and publication of data. Patient written consent for the publication of the study data was waived by the IRB because of the nature of the case series.

## Case One

**Figure 1:**
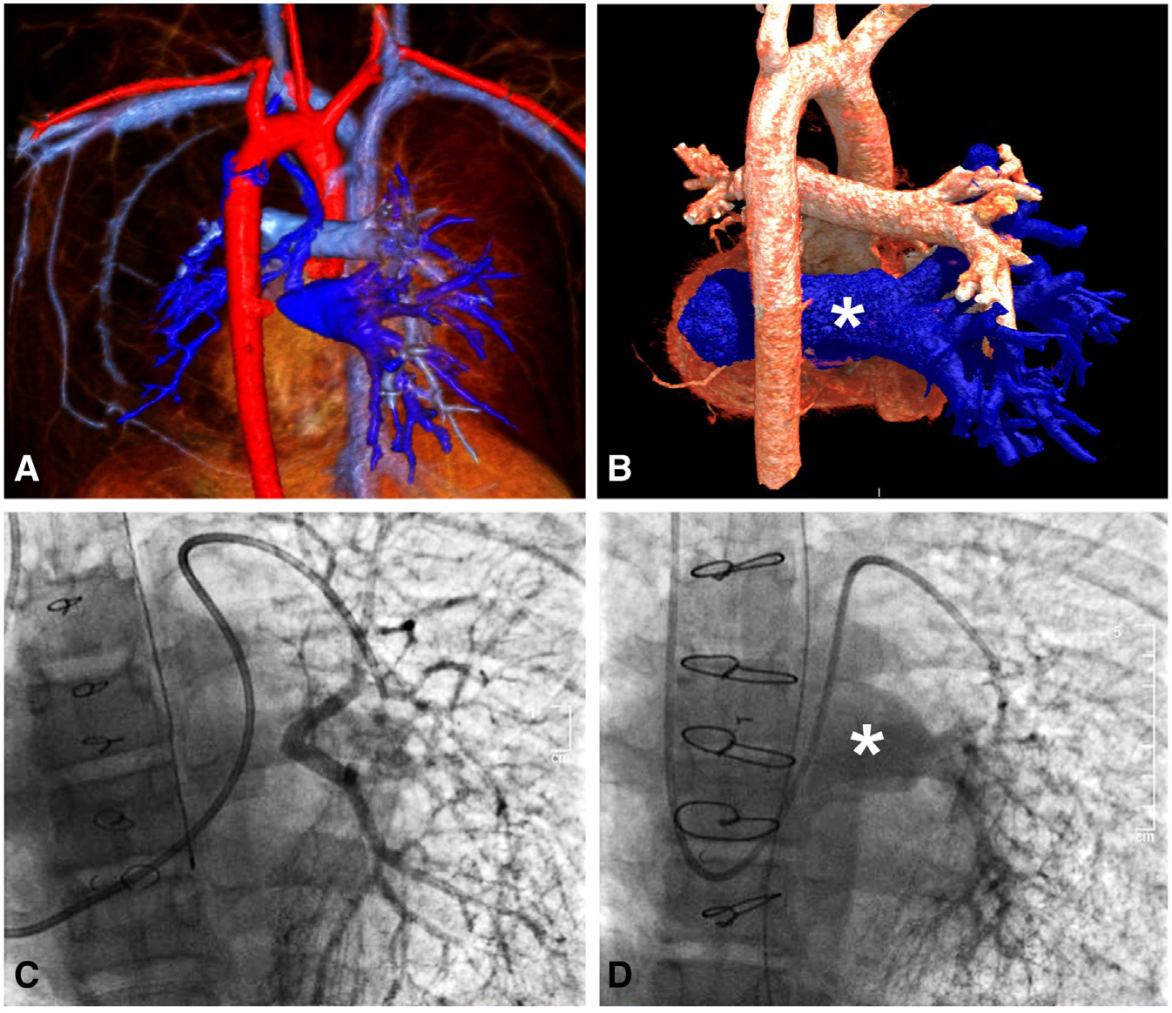
Case 1. A, Preoperative magnetic resonance imaging viewed from the posterior demonstrating the left superior and inferior pulmonary venous tributaries (*blue*) converging to a loose network of collaterals around the left hilum and peribronchial region. B, Postoperative computed tomography scan of the chest, 3-dimensional reconstruction, highlighting the pulmonary homograft (*blue*). C, Preoperative angiography demonstrating left PVO and D, postoperative angiography of left pulmonary veins and patent homograft (∗).

A 15-year-old patient with recurrent PVO of the left pulmonary veins previously underwent a re-repair with a sutureless technique and had received multiple balloon angioplasties of his pulmonary veins after standard repair of obstructed infradiaphragmatic TAPVR. Preoperative cardiac magnetic resonance imaging and catheterization (Figure 1, *A* and *C*) showed complete pulmonary venous obstruction outside of the lung parenchyma on the left. The left superior and inferior pulmonary venous tributaries converged to a loose network of collaterals around the left hilum and peribronchial region.

**Figure 2:**
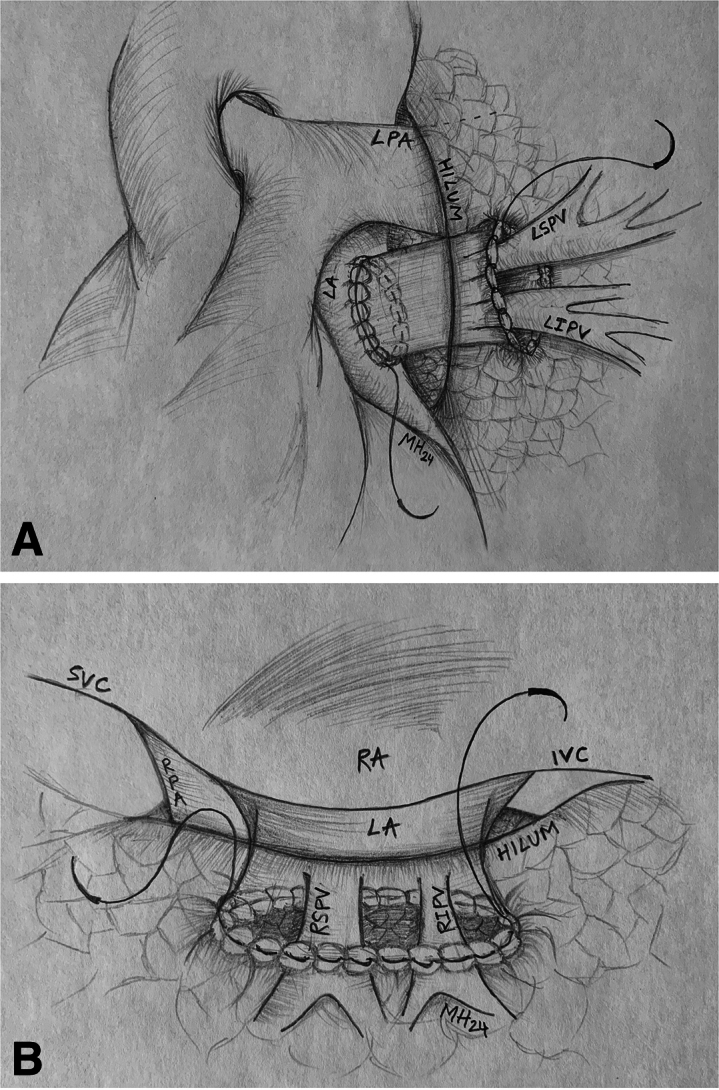
A, Kasai-type anastomosis of the muscular cuff of the pulmonary homograft to deflated lung tissue in the hilum and a sutureless-type anastomosis at the atrial level with free-floating pulmonary veins in the homograft. B, Kasai-type anastomosis of the left atrium directly to lung tissue in the hilum with free-floating pulmonary veins in the atrium. *LPA*, Left pulmonary artery; *LA*, left atrium; *LSPV*, left superior pulmonary vein; *LIPV*, left inferior pulmonary vein; *MH*, Majid Husain (illustrator); *SVC*, superior vena cava; *RA*, right atrium; *IVC*, inferior vena cava; *RPA*, right pulmonary artery; *RSPV*, right superior pulmonary vein; *RIPV*, right inferior pulmonary vein.

Intraoperatively, there was an atretic pulmonary vein cord attached to the left atrium (LA). This cord was dissected into the lung hilum until 2 small patent veins were visible. The atretic cord and thickened portion of the veins were excised to the level of maximum size for those particular veins. The distance between the hilum and LA was 4 to 5 cm, prohibiting any type of connection, especially precluding a primary sutureless connection to the atrium. Notably, there was no atrial appendage where the atretic attachment was identified, and a significant amount of pericardium had been previously dissected. Therefore, a 26-mm pulmonary homograft, with the valve excised, was used to bridge this gap. The anastomosis on the pulmonary side was a Kasai-type anastomosis. Specifically, the muscular cuff of the homograft was sewn to deflated lung tissue in the hilum adjacent to the pulmonary veins, and a sutureless-type anastomosis was performed at the atrial level. The lung was inflated to 30 cm of H_2_O pressure to ensure there was no air leak that could enter the connection (Figure 2, *A*).

The patient had an unremarkable postoperative course and was discharged on postoperative day 10. A computed tomography (CT) scan of the chest (Figure 1, *B*) before discharge showed laminar and unobstructed flow through the homograft into the LA. Catheterization at 3 months postoperatively (Figure 1, *D*) revealed continued patency of the left pulmonary veins. He remains asymptomatic and is doing well now 2 years postrepair.

## Case 2

**Figure 3:**
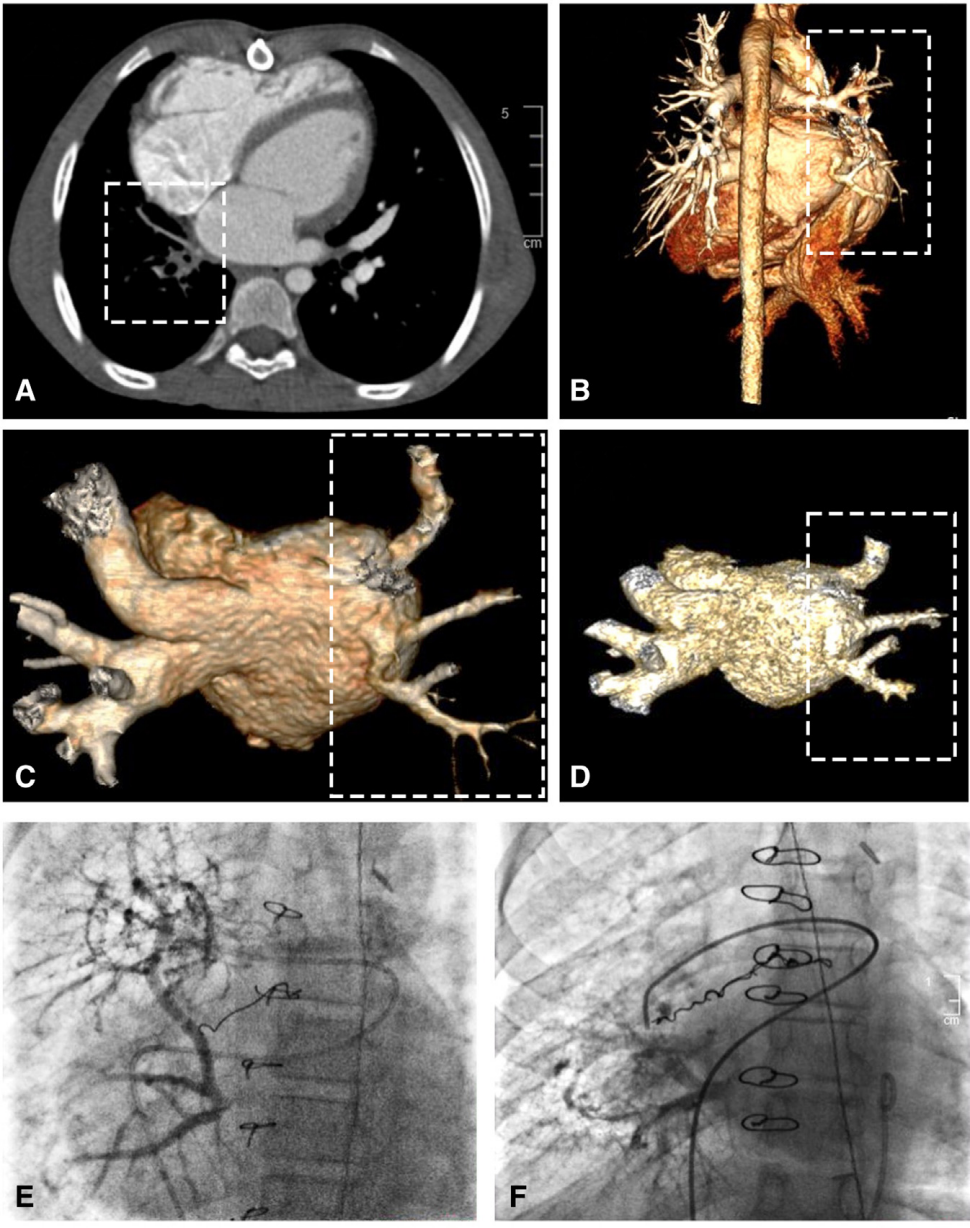
Case 2. A, Preoperative computed tomography scan of the chest demonstrating diminutive RPVs with no connection to the LA. B, Postoperative computed tomography scan of the chest, 3-dimensional reconstruction, demonstrating small-caliber RPVs. C, At 1-year follow-up and D, 2-year follow-up with progressively improving caliber. E, Preoperative angiogram demonstrating occluded RPVs but with distal patency and an extensive network of collaterals and F, postoperative angiography of right pulmonary veins after Kasai-type anastomosis of lung hilum to LA. *RPV*, Right pulmonary vein; *LA*, left atrium.

An 8-year-old with repaired obstructed infradiaphragmatic TAPVR and previous surgical revision for PVO as well as multiple catheterizations for coiling of arterial collaterals causing hemoptysis presented with recurrent PVO of the right pulmonary veins (RPVs). Preoperative CT angiogram (Figure 3, *A*) revealed diminutive RPVs with no connection to the LA. The RPVs were noted to course superiorly, beneath the right bronchus and carina, to join with mediastinal systemic veins. Preoperative catheterization (Figure 3, *E*) similarly revealed occluded RPVs but with distal patency and an extensive network of aortopulmonary and venovenous collaterals.

Intraoperatively, the occluded RPVs were identified. The atresia and occlusions were excised and dissected back to the hilum, where patent vein ostia were identified. A pericardial window was created adjacent to the hilum, and the 2 limbs of the RPVs were opened. A window was created in the LA and an anastomosis of the LA to the pericardium to the lung hilum was performed, thus connecting the hilum and free-floating pulmonary veins to the LA in a sutureless manner in some areas. In other areas, a portion of the lung was sewn directly to the left atrium. A Valsalva maneuver up to 30 mm Hg demonstrated no air leak into the repair (Figure 2, *B*).

The patient had phrenic nerve injury intraoperatively but had an unremarkable postoperative course and was discharged on postoperative day 4. CT angiogram at 1 year (Figure 3, *B* and *C*) and 2 years (Figure 3, *D*) postoperatively demonstrated improved caliber of the RPVs. Catheterization 3 years postoperatively revealed mild stenosis of the lower RPV; the upper and middle RPVs were widely patent (Figure 3, *F*). Four years postrepair, she continues to do well.

## Discussion

PVO is a major complication and the main cause of reoperation after repair of TAPVR.^1^^,^^2^ Use of the standard sutureless technique to address this pathology may not be possible when patent pulmonary veins are relatively remote from the atrium or well outside of the pericardial reflection. To overcome this hurdle, we have demonstrated a lung hilum Kasai-like anastomosis, of either the atrium or a conduit, to be a variant of some utility with medium-term durability.

Others have reported the use of an in situ pericardial roll technique to repair variants of anomalous venous return where the pulmonary veins were distant from the LA.^4^ A modified Warden operation using a femoral vein homograft to repair drainage of right-sided pulmonary veins to the azygos vein, a rare variant of anomalous pulmonary veins has also been described.^5^

Exploration and implementation of innovative techniques to address this persistent and recurring pathology is paramount in reducing its associated morbidity and mortality. Our cases highlight such surgical repairs to address these challenges and adds to the repertoire of the surgeon.

## Conflict of Interest Statement

The authors reported no conflicts of interest.

The *Journal* policy requires editors and reviewers to disclose conflicts of interest and to decline handling or reviewing manuscripts for which they may have a conflict of interest. The editors and reviewers of this article have no conflicts of interest.
